# Supplementary material for: Short- and long-term memory of moving amoeboid cells
Source: PLoS One. 2021 Feb 11;16(2):e0246345. doi: 10.1371/journal.pone.0246345 (PMC7877599; doi:10.1371/journal.pone.0246345)
Supplement: S1 Fig — (PDF) [file pone.0246345.s001.pdf]

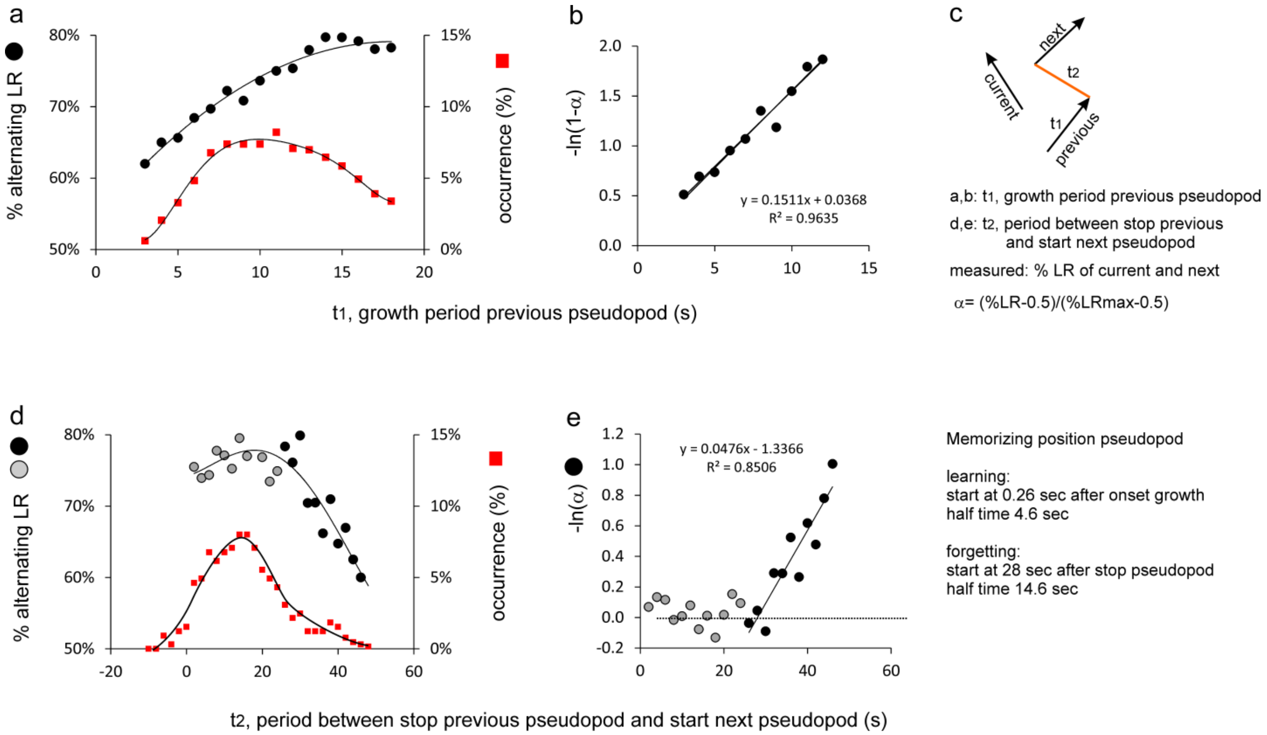

Figure S1. Kinetics of positional memory, defined by %LR. In series of splitting pseudopods the direction of the pseudopod relative to the previous pseudopod was determined as Left or Right. Series of three pseudopods were identified as LR, RL, LL or RR. The % LR is the percentage of LR+RL. The %LR was measured for different growing times of pseudopod P1 (learning time t1 in panels a, b) and for different intervals between stop of P1 and start of P3 (forgetting time t2 in panels d, e). Panels b and e are logarithmic transformation of the original data; the data points represented by the black bullets in panels d and e are incorporated in the linear regression. The kinetic constants for learning and forgetting were obtained by linear regression analysis and show the optimal fit ± 95% confidence levels. See Figure 2 for a similar analysis using cos(ϕ1,3) instead of %LR.
