# Supplementary material for: Short- and long-term memory of moving amoeboid cells
Source: PLoS One. 2021 Feb 11;16(2):e0246345. doi: 10.1371/journal.pone.0246345 (PMC7877599; doi:10.1371/journal.pone.0246345)
Supplement: S2 Fig — (PDF) [file pone.0246345.s002.pdf]

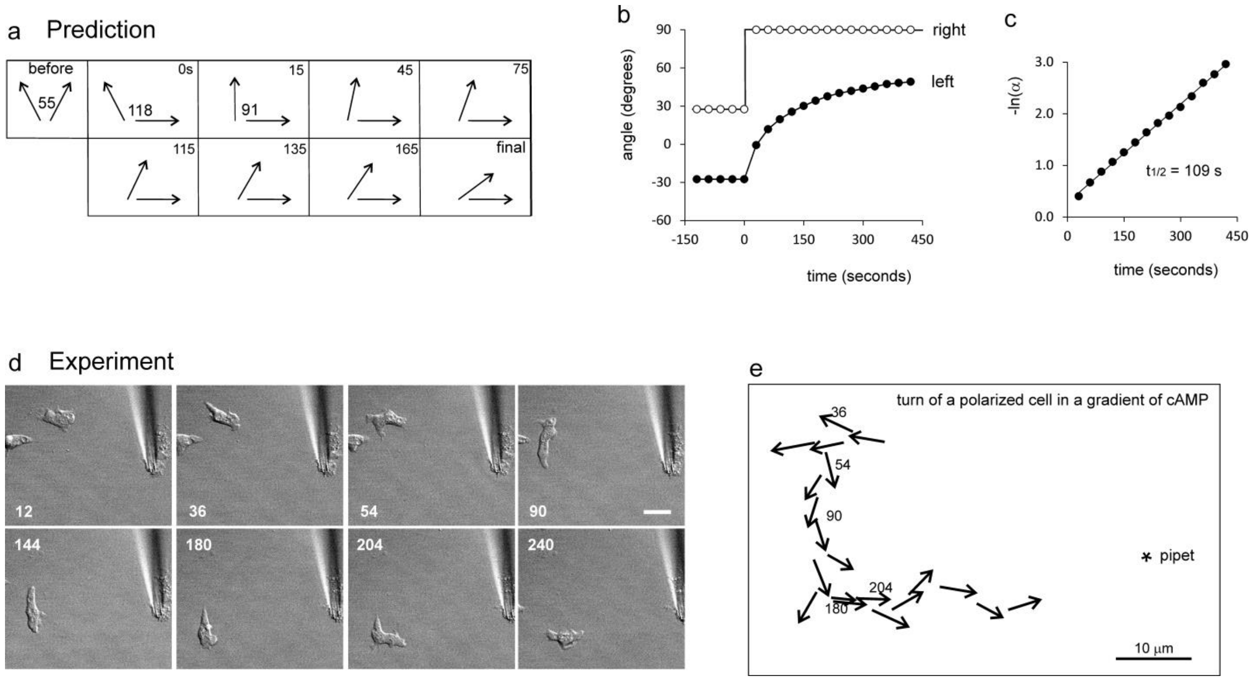

Figure S2. The kinetics of turning in buffer and in a chemotactic gradient.
Panels abc: buffer. Figure 8ab revealed the relationship between the change of direction of a new pseudopod and the change of the polarity axis. at the polarity axis . a. Prediction of cell movement based on the polynomial fit of the data of Fig. 8ab. A cell is moving at 0 degrees with alternating Left/Right pseudopods at an angle of 55 degrees, thus Left pseudopod at -27.5 degrees and Right pseudopod at +27.5 degrees. From t = 0 s all Right pseudopods are extended to the West. The experimental information of the green area was used to calculate the direction of the Left pseudopods. b. The angles of the Left and Right pseudopod. c. The change of the direction of the Left pseudopod was analyzed by logarithmic transformation of the data, with a = 0.00636 s^{-1}. The results show that the cell slowly turns with a half time of about 110 seconds.
Panels de: chemotaxis. Starved *Dictyostelium* cells are exposed to a micropipette filled with 0.1 μM cAMP. d. Phase contrast images; the numbers indicate the time in seconds after application of the cAMP gradient. The cell indicated by the arrow was analyzed. e. Pseudopod analysis of the cell; each arrow is a pseudopod. Initially the cell indicated by the arrow moves with a few pseudopods against the gradient, makes a 90 degrees turn at 54 seconds followed by a few pseudopods in the same direction, and makes again a 90 degrees turn at 180 seconds to move in the direction of the pipet. The experiment was repeated 5 times, yielding a half-time of turning in a chemical gradient of 130 ± 25 seconds (mean and SD).
