# Supplementary material for: Short- and long-term memory of moving amoeboid cells
Source: PLoS One. 2021 Feb 11;16(2):e0246345. doi: 10.1371/journal.pone.0246345 (PMC7877599; doi:10.1371/journal.pone.0246345)
Supplement: S3 Fig — (PDF) [file pone.0246345.s003.pdf]

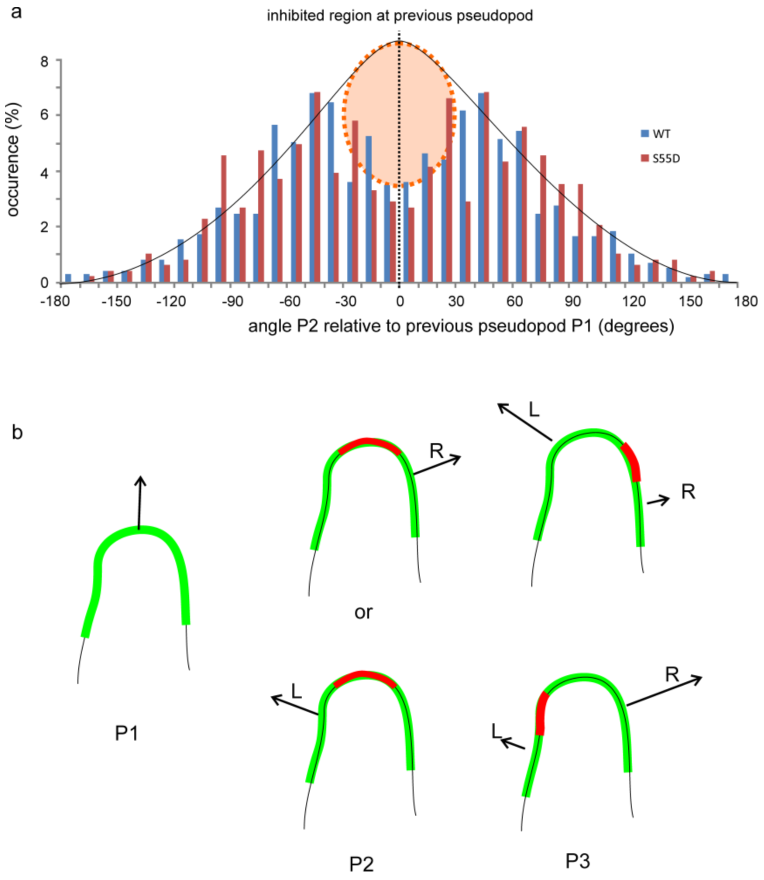


**Figure S3. Polarized cells without positional memory have a small alternating L/R bias.** Panel a shows the extension of Pseudopod P2 relative to the position of pseudopod P1 for wild-type and mutant Scar-S55D cells. The data reveal that P2 rarely is extended in the same direction as P1; P1 leaves behind an area of inhibition for P2 that is about 30 degrees . Panel b) shows a schematic of pseudopod extensions in polarized cells without positional memory. Green, activated front due to polarity, red inhibited area due to previous pseudopod. This cell has no history, so P1 is assumed to be extended along the polarity axis. This leaved behind an inhibited area, by which P2 is extended either at the Right side (top) or Left side (bottom). Discussion now follows for the top panel, bottom panels lead to the same conclusion. In the top panel the area of inhibition that is left behind by P2 is present at the Right side of the front. As a result, the green activated area at the Right side is smaller than at the Left side, making a Left pseudopod more likely than a Right pseudopod, thereby inducing a bias of increased alternating L/R. Based on the experimental data of panel a, the length of the activated green area is about 60 degrees at the Right and about 90 degrees at the Left, predicting a %LR = ~60% for polarized cells without positional memory. For unpolarized cells the length at the left is 180 degrees and at the right 150 degrees, predicting %LR = ~54% for unpolarized cells without positional memory. The observed experimental data are: Starved wild-type with polarity and position memory: %LR = 77±4; starved mutant Scar-S55D with polarity memory but no positional memory; %LR =56±5; vegetative mutant Scar-S55D with neither polarity memory nor positional memory; %LR =56±3.
