# Supplementary material for: Short- and long-term memory of moving amoeboid cells
Source: PLoS One. 2021 Feb 11;16(2):e0246345. doi: 10.1371/journal.pone.0246345 (PMC7877599; doi:10.1371/journal.pone.0246345)
Supplement: S4 Fig — (PDF) [file pone.0246345.s004.pdf]

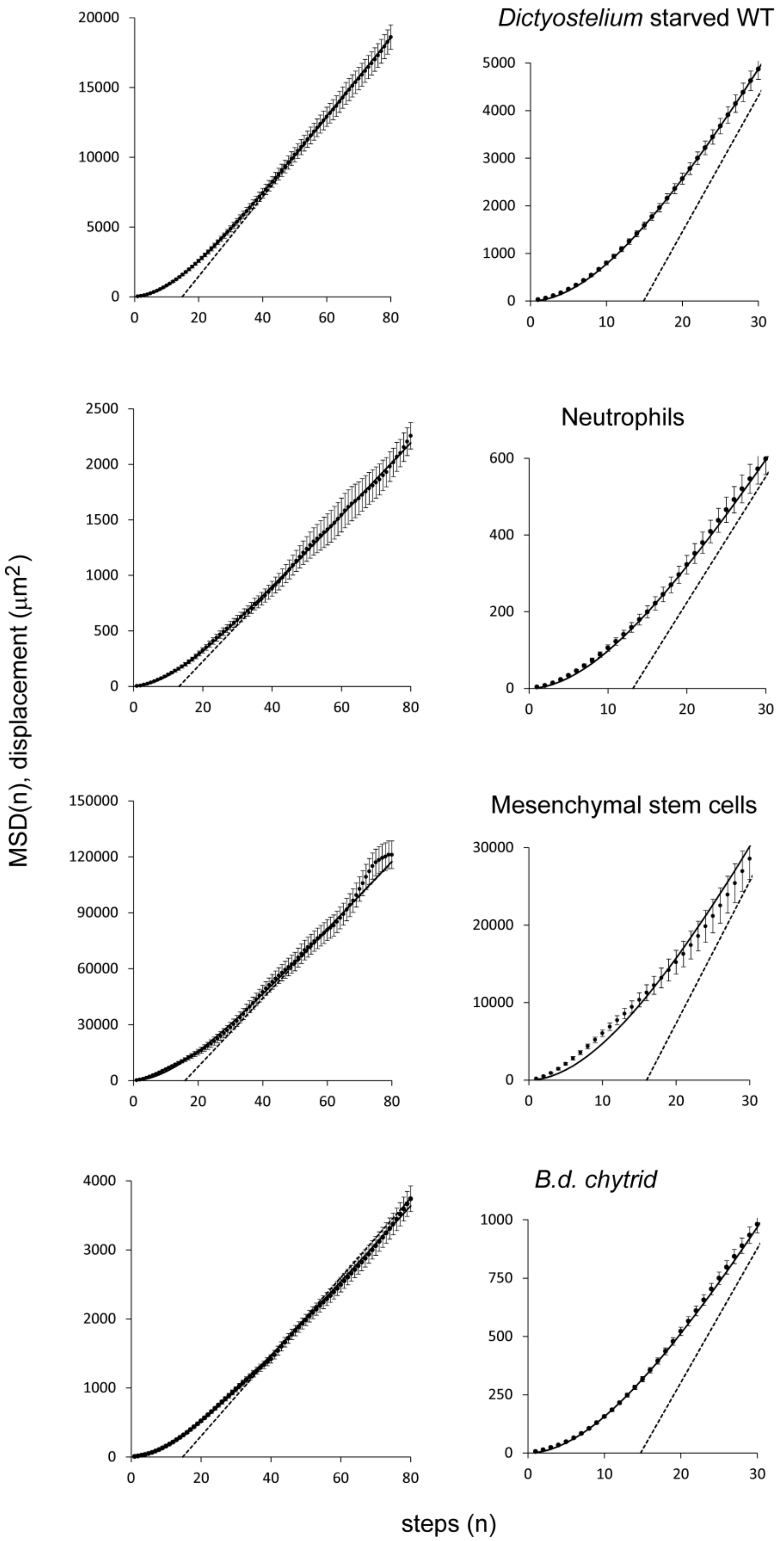


***Figure S4. Persistence; part 1 of 3. Dictyostelium starved wild-type and three other species.***

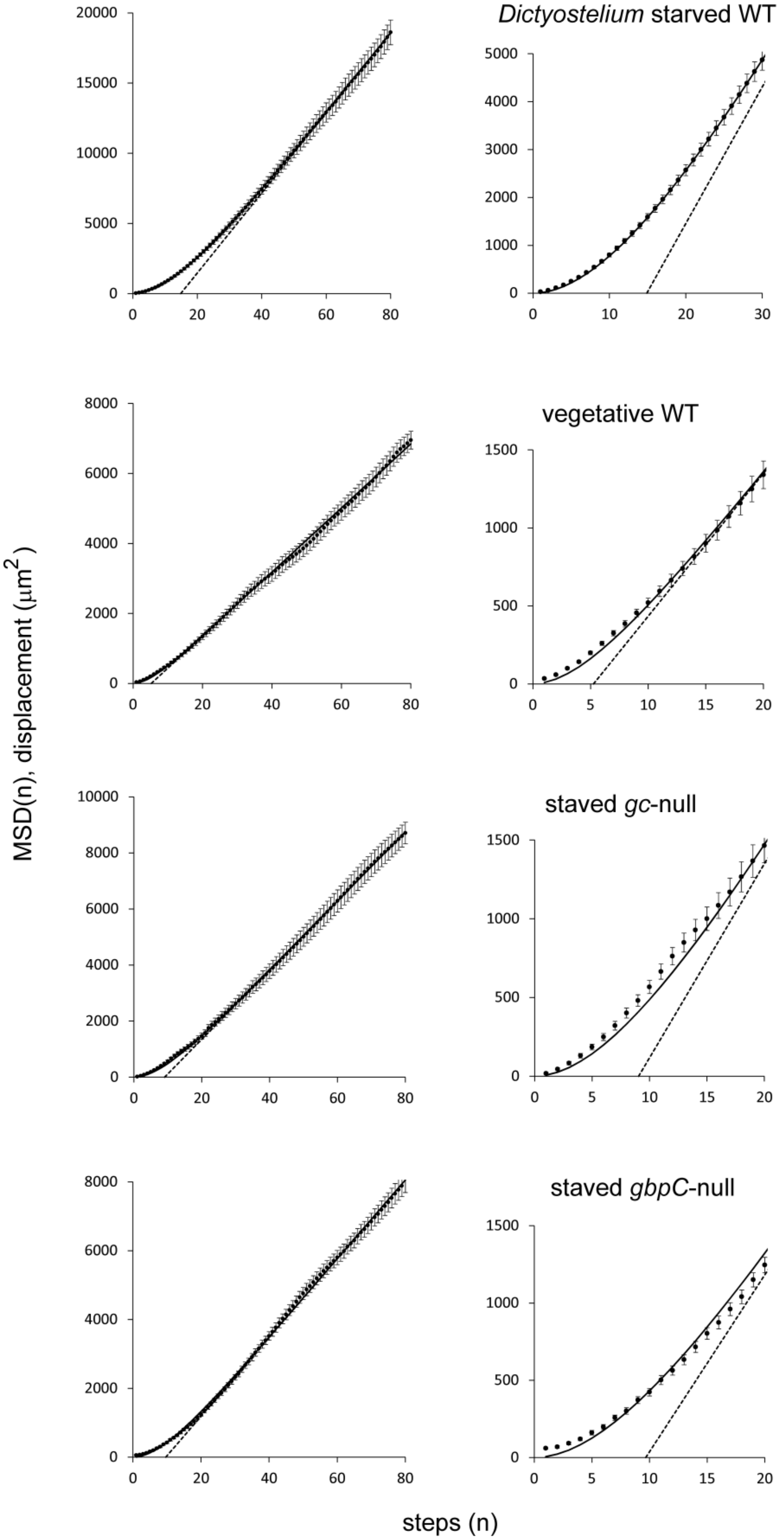


***Figure S4. Persistence; part 2 of 3. Dictyostelium starved wild-type, vegetative cells and mutants.***

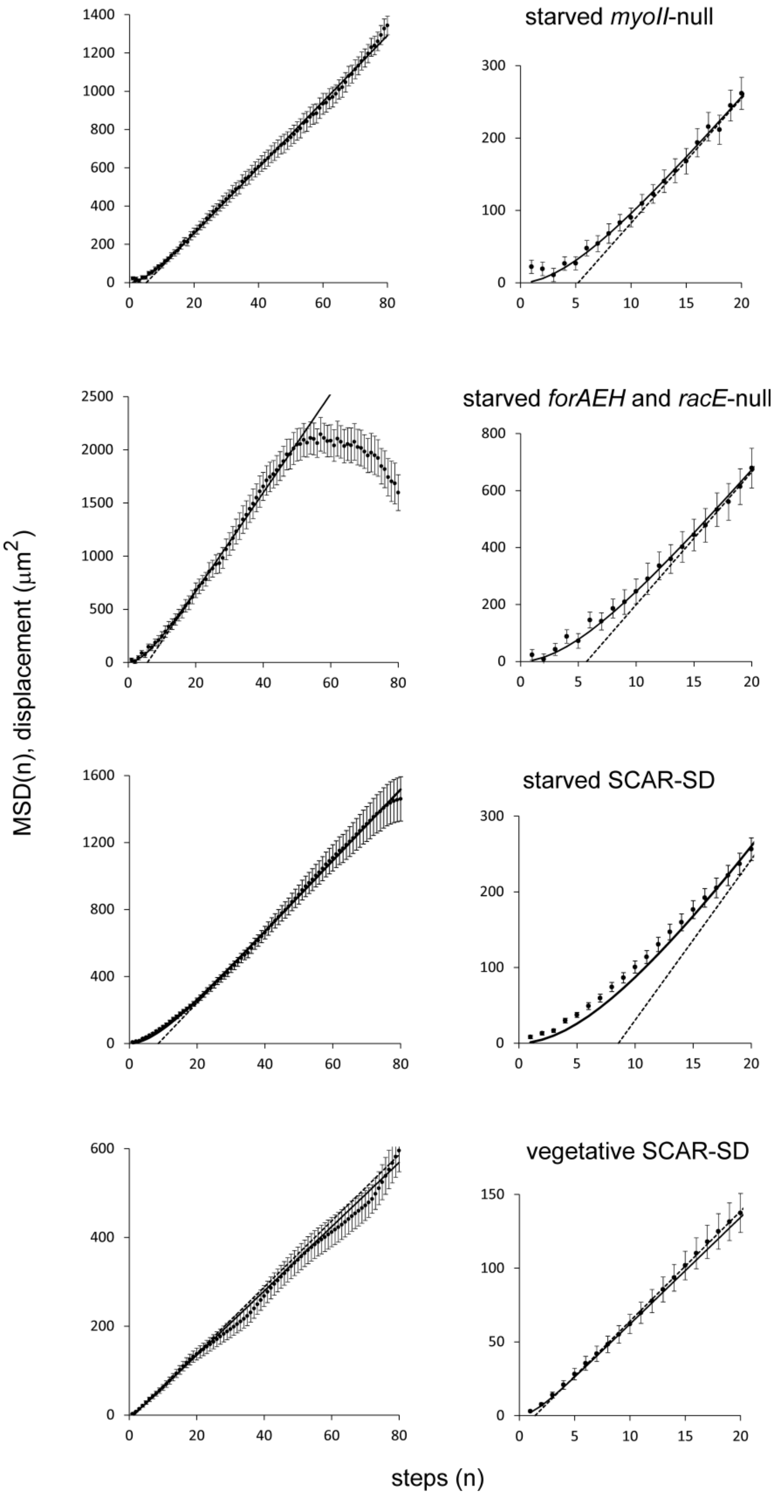


***Figure S4. Persistence; part 3 of 3.*** Figures show the MSD as function of n, the number of steps. The data are the means and 95% CI. The solid line is the optimal fit using equation (1), yielding estimates for the step size λ and the persistence P (see table 1 and table S1). The dashed line is the asymptote for large n; the intersection with the x-axis is the persistence P. For mesenchymal stem cells data up to n=70 and for *forAEH*/*RacE* cells data up to n=50 were used for the fit.
