# Supplementary material for: Short- and long-term memory of moving amoeboid cells
Source: PLoS One. 2021 Feb 11;16(2):e0246345. doi: 10.1371/journal.pone.0246345 (PMC7877599; doi:10.1371/journal.pone.0246345)
Supplement: S5 Fig — (PDF) [file pone.0246345.s005.pdf]

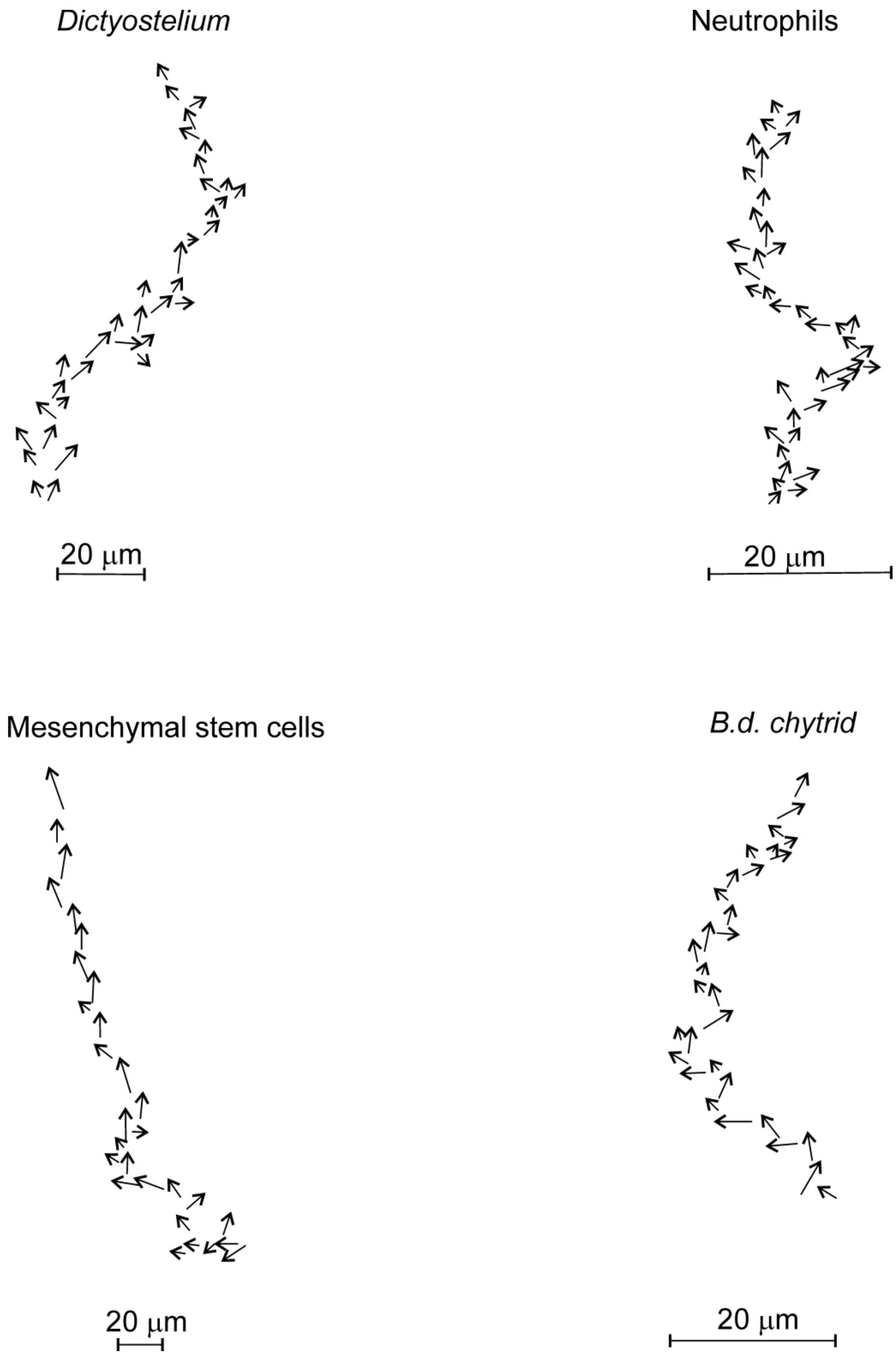

Figure S5. Path of four cell lines. The arrows indicate the pseudopods. The total time is 9.5 min for *Dictyostelium* staved wild-type, 5 min for neutrophils, 10 hours for mesenchymal stem cells, and 3.5 min for *B.d. chytrid*.
