# Supplementary material for: Short- and long-term memory of moving amoeboid cells
Source: PLoS One. 2021 Feb 11;16(2):e0246345. doi: 10.1371/journal.pone.0246345 (PMC7877599; doi:10.1371/journal.pone.0246345)
Supplement: S1 Table — (PDF) [file pone.0246345.s006.pdf]

Supporting information to: Short- and long-term memory of moving amoeboid cells, by Peter J.M. Van Haastert

Table S1. Persistence data.

	primary pseudopod data				mean square displacement (MSD)				calculated	
	size (µm)		interval (s)		λ, step (µm)		P, steps		P, time (min)	
strain	mean	95% CI	mean	95% CI	opt. fit	95% CI	opt. fit	95% CI	mean	95% CI
Starved WT	5.37	0.13	15.42	0.71	3.09	0.09	14.90	0.96	3.83	0.30
Vegetative WT	4.90	0.18	16.89	1.39	2.83	0.49	5.72	1.64	1.61	0.48
Starved *gc*-null	4.64	0.23	11.17	1.19	2.61	0.36	9.05	1.88	1.68	0.39
Starved *gbpC*-null	5.02	0.27	9.46	1.13	2.43	0.18	9.68	1.09	1.53	0.25
Starved *myoII*-null	5.02	0.22	14.63	1.56	1.29	0.30	5.21	1.85	1.27	0.47
forAEH/*racE*-null	3.89	0.24	5.87	1.09	2.02	0.47	5.72	2.18	0.56	0.24
Starved Rap1G12V	3.06	0.16	9.21	1.11	n.d.	n.d.	n.d.	n.d.	n.d.	n.d.
Starved SCAR-SD	2.86	0.12	8.70	1.87	1.11	0.01	8.57	1.03	1.24	0.31
Vegetative SCAR-SD	2.63	0.28	6.42	1.02	1.61	0.32	1.43	0.39	0.15	0.05
Neutrophils	3.32	0.21	7.86	1.07	1.12	0.13	13.16	1.61	1.72	0.32
Mesenchymal stem	13.80	0.98	1289	206	7.56	0.92	16.01	2.64	344	79
B.d. chytrid	2.21	0.13	7.26	0.77	1.40	0.04	14.74	0.57	1.78	0.20

Primary pseudopod data are derived from Van Haastert (2020) PLoS One e0243442, or obtained here for SCAR-SD. The MSD data of figures S4 were used to estimate the step size λ in µm and the persistence P in steps; the 95% confidence interval (CI) was obtained by bootstrap analysis of the data. The Persistence in time is calculated using the pseudopod interval and the persistence in steps. Note the extreme large persistence time of 344 minutes for mesenchymal stem cells, which is due to the very large pseudopod interval (1289 seconds).
