# Supplementary material for: Short- and long-term memory of moving amoeboid cells
Source: PLoS One. 2021 Feb 11;16(2):e0246345. doi: 10.1371/journal.pone.0246345 (PMC7877599; doi:10.1371/journal.pone.0246345)
Supplement: S2 Table — (PDF) [file pone.0246345.s007.pdf]

Supporting information to: Short- and long-term memory of moving amoeboid cells, by Peter J.M. van Haastert

Table S2. Source of movies.

strain	reference	remarks
WT, polarized	[1–4]	main source of movies
	[5–7]	WT control of mutant studies
WT, unpolarized	[3,5,8]	
SCAR-SD in *scar*-null	[5]	movies used here
		see also Ura et al [9]
pla2-null	[2]	
gc-null	[2]	
gbpC-null	[6,10]	
myoII-null	[5]	
Rap1G12V	[1,11]	
forAEH-null	[12]	
racE-null	[12]	
lrrA-null	[13]	
Neutrophils	[14]	
	[15]	movie from the Babraham Institute; downloaded on May 6, 2015.
Mesenchymal stem cells	[16]	movie from the Institut fur Technische Chemie, Hannover; downloaded on May 6, 2015.
		reference to authors of the movie [17]
B.d. chytrid	[18]	

WT: wild-type *Dictyostelium*

References

1. Van Haastert, P.J.M. (2020). Unified control of amoeboid pseudopod extension in multiple organisms by branched F-actin in the front and parallel F-actin/myosin in the cortex. PLoS One *15*, e0243442.
2. Bosgraaf, L., and Van Haastert, P.J.M. (2009). The ordered extension of pseudopodia by amoeboid cells in the absence of external cues. PLoS One *4*, e5253.
3. Van Haastert, P.J.M., and Bosgraaf, L. (2009). Food searching strategy of amoeboid cells by starvation induced run length extension. PLoS One *4*, e6814.
4. Bosgraaf, L., and Van Haastert, P.J.M. (2009). Navigation of chemotactic cells by parallel signaling to pseudopod persistence and orientation. PLoS One *4*, e6842.

5. van Haastert, P.J.M., Keizer-Gunnink, I., and Kortholt, A. (2018). The cytoskeleton regulates symmetry transitions in moving amoeboid cells. J. Cell Sci. *131*.
6. Kortholt, A., van Egmond, W.N., Plak, K., Bosgraaf, L., Keizer-Gunnink, I., and van Haastert, P.J.M. (2012). Multiple regulatory mechanisms for the Dictyostelium Roco protein GbpC. J. Biol. Chem. *287*, 2749–58.
7. Van Haastert, P.J.M., and Bosgraaf, L. (2009). The local cell curvature guides pseudopodia towards chemoattractants. HFSP J. *3*, 282–6.
8. Van Haastert, P.J.M. (2010). A model for a correlated random walk based on the ordered extension of pseudopodia. PLoS Comput. Biol. *6*.
9. Ura, S., Pollitt, A.Y., Veltman, D.M., Morrice, N.A., Machesky, L.M., and Insall, R.H. (2012). Pseudopod growth and evolution during cell movement is controlled through SCAR/WAVE dephosphorylation. Curr. Biol. *22*, 553–561.
10. Veltman, D.M., and van Haastert, P.J.M. (2008). The role of cGMP and the rear of the cell in Dictyostelium chemotaxis and cell streaming. J. Cell Sci. *121*, 120–127.
11. Plak, K., Keizer-Gunnink, I., van Haastert, P.J.M., and Kortholt, A. (2014). Rap1-dependent pathways coordinate cytokinesis in Dictyostelium. Mol. Biol. Cell *25*, 4195–4204.
12. Litschko, C., Bruhmann, S., Csiszar, A., Stephan, T., Dimchev, V., Damiano-Guercio, J., Junemann, A., Korber, S., Winterhoff, M., Nordholz, B., *et al.* (2019). Functional integrity of the contractile actin cortex is safeguarded by multiple Diaphanous-related formins. Proc. Natl. Acad. Sci. U. S. A. *116*, 3594–3603.
13. Kamp, M. (2019). Regulation of G-protein during chemotaxis in space and time. PhD thesis, Groningen Univ.
14. Itakura, A., Aslan, J.E., Kusanto, B.T., Phillips, K.G., Porter, J.E., Newton, P.K., Nan, X., Insall, R.H., Chernoff, J., and McCarty, O.J.T. (2013). p21-Activated kinase (PAK) regulates cytoskeletal reorganization and directional migration in human neutrophils. PLoS One *8*, e73063.
15. https://www.youtube.com/watch?v=qOX5Z7AutM4.
16. https://www.youtube.com/watch?v=RRUvRJNlW84.
17. Kirsch, M., Birnstein, L., Pepelanova, I., Handke, W., Rach, J., Seltsam, A., Scheper, T., and Lavrentieva, A. (2019). Gelatin-Methacryloyl (GelMA) Formulated with Human Platelet Lysate Supports Mesenchymal Stem Cell Proliferation and Differentiation and Enhances the Hydrogel's Mechanical Properties. Bioeng. (Basel, Switzerland) *6*.
18. Fritz-Laylin, L.K., Lord, S.J., and Mullins, R.D. (2017). WASP and SCAR are evolutionarily conserved in actin-filled pseudopod-based motility. J. Cell Biol. *216*, 1673–1688.
