# Supplementary material for: Short- and long-term memory of moving amoeboid cells
Source: PLoS One. 2021 Feb 11;16(2):e0246345. doi: 10.1371/journal.pone.0246345 (PMC7877599; doi:10.1371/journal.pone.0246345)
Supplement: Attachment — Submitted filename: R2R 26226R1.pdf [file pone.0246345.s008.pdf]

Rebuttal letter

Dear Thierry,

Thank you for handling this manuscript and the possibility to submit a revised version of the manuscript.

I thank the reviewers for their positive and instructive comments. This manuscript deals with a rather unusual and detailed analysis of cell movement. I am aware that this is not easy material for many cell biologist. The changes made in response to the reviewers comments have made the paper considerably more accessible to the readers. In response to several question of the reviewers on explanation of terms such as persistence, memory and random, I have included a section in the revised manuscript with a box of nomenclature, definition and terms. Where possible, I have followed all suggestions of the reviewers. Major changes are a detailed analysis of cell trajectories and persistence, and two supplementary tables added. Since in the revised manuscript the symbol P is used for persistence, the symbol P for pseudopod activity had to be modified; chosen was the Greek letter Π.

The revised manuscript (marked track changes) has the changed annotated yellow for large pieces of changed text, and as track-changes for small pieces of text. Changes in the reference list are not marked.

I hope that the manuscript is now acceptable for publication in PLoS One.

With kind regards,

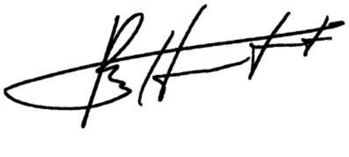

Peter van Haastert

Response to the reviewers

Reviewer #1: In this work, the author aims to describe the mechanism underlying persistent migration of dictyostelium cells. To do so, he relates the migration of the cells to the dynamics of pseudopods. He then aims at connecting the pseudopod dynamics to signaling pathways. I have two main problems with the manuscript:
1. Unfortunately, the author uses the language of memory to describe his findings. I found this utterly confusing, because it suggests some separate 'module' that stores the orientation of the polarization axis. In what he describes, however, this 'memory' is given by the elements that define the polarity axis; that is the pseudopods. This language made it very hard for me to differentiate between observations and (proposed) mechanisms. Also the level of description changes: sometimes the focus is on the molecular players, sometimes on the pseudopods and I could not decipher, where the mechanism lies. Eventually, I think, that 'memory' is just another word for 'persistence'. I prefer the latter as it is much less prone to confusion.

In the revised manuscript I have added a box with nomenclature, definition and terms used. It also contains a description of the difference between memory and persistence: memory is an essential fundamental component of a correlated random walk to provide correlation of directional movement, while persistence is an experimental observable describing the length of the correlation detectable. The text has been modified carefully to follow these definitions.

2. The author has a tendency to base claims about mechanisms on correlations.

The manuscript was changed to avoid strong claims based only on correlations, such as on pages 9, 10 and 16.

3. The model defined by Eq.(1) remained obscure to me: what is the inactive axis? What defines this axis? Why does it not appear in the equations? Suddenly, in the methods part on p. 25, the authors speaks about old and new axes that have not been (properly) introduced in this modeling section. How do they interact, if at all? How are they oriented? Their relative orientation is seemingly unimportant. Why?

The model defined in Eq. (2) is a conceptual model of the polarity axis. In the experiments, the molecules that define the polarity axis include cGMP-occupied GbpC and myosin in the contractile cortex. Therefore, the concept of an inactive polarity axis can be understood for instance as the unoccupied GbpC or a weak contractile cortex that allow to make new pseudopods in the rear of the cell. This is described on page 17 of the revised manuscript.

The model is a kinetic model to uncover the half-times of formation of a polarity axis and the half-times of loss of the polarity axis. The model does not describe the direction or orientation of the polarity axis. The activity of the axis depends on pseudopod activity.

The new and old axes are introduced in the model part on page 27 of the revised manuscript.

Since many points remained unclear to me, I cannot recommend publication of this work in its current state.

Specific comments:

Abstract: “The direction of movement is not random, but is correlated with the direction of movement in the preceding minutes” There is no logical connection between the two parts of the sentence: randomness and correlation in the direction of movement are independent features. Notably, a movement can be random and still be correlated with the movement before. A classic example of such a behavior is a persistent random walk, described by Fürth 100 years ago. The author knows this as indicated by the first paragraph of the introduction, where he writes about “correlated random walks”. But then he contradicts hjmself by writing “persistence versus random movement”. Again, these two notions are not mutually exclusive.

This specific comments suggests that the reviewer’s field of science uses a very different perspective of the terms ‘random’, ‘persistence’, ‘correlated’, and ‘memory’ than used in the manuscript. Therefore, I have added in the revised manuscript a box with explanations, nomenclature, definitions and terms.

My perception is the following: In a true random walk the next step is not correlated with the previous step in any sense, not length, not direction, not timing. A correlated random walk is not random, BECAUSE the next step is -in some way- correlated with the previous step in length, direction or timing. The word random in correlated random walk is confusing (and in my perception not correct): a walk is either random, or it is correlated and then not truly random anymore. I understand the origin of the term ‘correlated random walk’: theoretically it describes a random walk with added correlation, and experimentally the walk appears as a random walk with a little bit of correlation that is statistically significant. In my perception, a clear distinction between a random walk and a correlated random walk is fundamentally important, because a correlated random walk must have a molecular mechanism that allows for the correlation, while a true random walk does not need to have such a mechanism. This molecular mechanism is called memory by me and many others; a random walk does not need to have memory. And it is this memory that I want to identify and characterize at a molecular level in this manuscript. This line of thought is the basis of the information provided in the box of the revised manuscript.

P. 3, 2nd paragraph: “The timing and direction of pseudopod formation in amoeboid cells has been described as an ordered stochastic process” This process has also been described as resulting from deterministic dynamics: Dreher et al NJP 2014, Stankevicins et al, PNAS 2020

I am not familiar with deterministic dynamics, but I understand from Wikipedia that “in a system with deterministic dynamics randomness is NOT involved in the development of future states of the system. A deterministic model will thus always produce the same output from a given starting condition or initial state.” I feel that this is not what I want to express in pseudopod extension, which has substantial randomness with underlying order. Monte Carlo simulations using the

observed order and variation predict very different trajectories that originate from the same starting conditions (reference 10).

P. 3, 2nd paragraph: “this zig-zag trajectory provides persistence” Persistence is formally defined via the direction autocorrelation, which typically decays exponentially. The characteristic decay time is the persistence time. For an “ideal” zig-zag trajectory, the direction autocorrelation would be a periodic function. So, I am not sure what the author wants to say.

Dictyostelium cells make de novo pseudopods in random directions and splitting pseudopods in the front alternatingly to the right and left, in a ratio of one de novo and between four to ten splitting. The autocorrelation of a zig-zag trajectory of stretches of only splitting pseudopods has been shown to be a periodic function that decays with time (reference 7), as predicted by the reviewer. In the revised manuscript it is now mentioned that a zig-zag trajectory contributes to the observed persistence.

P/4, 1st paragraph: here the author introduces the notion of memory. It was not clear to me, whether memory was used as a synonym for persistence or whether this was a new concept. Since memory is used in different contexts with different meanings, I invite the author to clarify. If it is the former, I would suggest to not mention the word memory at all.

Persistence is the observation that the correlation of direction decays with time. Memory is the underlying molecular mechanism that can store directional information. This has been defined in the box of the revised manuscript.

P.5, 1st paragraph: “the typical properties of a correlated random walk with a correlation/persistence of about 11 pseudopods, representing the memory of this cell (Fig. 1b). The sequence of three pseudopods is frequently alternating to the left and right, contributing to a persistent zig-zag trajectory” Just to repeat my previous comments: the same words are used in different contexts, which is confusing. Please, define clearly what you mean and then stick to your defined terms. - The same problem is visible throughout the manuscript and should be rectified.

Definitions for persistence and memory are given in the revised manuscript in the new box with nomenclature, definitions and use of terms.

P.6, last paragraph: “the periodic autocorrelation time is about twice the pseudopod interval. This means that the start of the first pseudopod is correlated with the start of the third pseudopod, while the start of the second pseudopod is correlated with the fourth pseudopod.” Agreed, but this does not discriminate between the two mechanisms, that is, whether the direction of P3 is determined by the position of P1 or P2. In fact there is a strong anti-correlation at about 15 s, which suggests that P2 is determined by P1 (and P3 by P2), if I follow the argument of the author.

Previously we have shown that a new pseudopod P2 cannot immediately start at the position where the previous pseudopod P1 stopped. Therefore, P2 must start elsewhere explaining the anti-correlation.

p. 7: “The kinetics of learning and forgetting the position of splitting pseudopods” I really do not understand, why I should invoke the word learning in this context. If the cell just deposited a marker at the place where a pseudopod is formed, would you call this learning?

This is called learning, because it takes time -and for the polarity axis several repeated depositions by multiple pseudopods- to establish memory.

p. 9, 2nd paragraph: “pseudopod activity in the front is the input signal to generate, memorize and maintain a polarity axis.” From a correlation you infer a mechanism, but you do not have any evidence for a causal relation between pseudopod activity and persistence.

There are two pieces of evidence: First, the observation that cells with stalled pseudopods in the front make pseudopods at the side and loose persistence. Second, inhibition of F-actin that is largely formed in pseudopods leads to reduced cGMP levels, which is known to suppress pseudopods at the side. From this evidence I propose that pseudopod activity in the front is a positive input signal to establish a polarity axis.

P. 10, 1st paragraph: “It should be noted that cGMP diffuses rapidly in the cell [28], and that cGMP induces an increase of myosin filament formation in the entire cell [17]. However, cell polarity is associated with increased myosin filaments in the rear and reduced myosin filaments in the front of the cell [29].” How are these two sentence are meant to be understood? As an introduction to the rest of the paragraph; as pointing out a contradiction?

This is an introduction to indicate that cGMP is required but not sufficient, that is explained in the rest of the paragraph with activation of Rap in the front that inhibits myosin filaments. This has been added on page 10 of the revised manuscript.

p. 11, 2nd paragraph: “Produced cGMP rapidly diffuses in the cell, activates GbpC leading to an increase of myosin filament in the entire cell. The second pathway also starts with Ras-GTP in the front and mediates the activation of Rap1 in the front half of the cell.” If I understood correctly, you have tracked the distributions of various proteins, but not their activities. How can you thus draw these conclusions about the spatiotemporal activation profiles?

The facts are that cGMP is produced (not known where), cGMP diffuses rapidly (measured), cGMP activates GbpC (measured in cells; not known where), and this leads to myosin formation the entire cell (measured). The facts for the other pathway are: active Ras-GTP is formed in the front (measured with specific sensors that detect specifically Ras-GTP), Rap1 is also activated in the front (measured with specific sensors that detect specifically Rap1-GTP), and Rap1 activation depends on Ras activation (established with mutants). So it appears that everything is consistent.

p. 11, last paragraph: The way the model is referred to seems to me too vagua. The model should

at least roughly be explained in the main text: what are the main assumptions, how are the assumptions implemented, how is the model analyzed?

The text at the end of polarity (page 11) combines the experimental observation on the kinetics of formation and loss of the polarity axes, the molecules involved and the localization of their formation. It is not a formal model but a summary of the previous sections on polarity.

p. 12, 1st paragraph: "For determining exact kinetics, only those 28 cases were used in which the Old front does not exhibit any pseudopod activity after the New front was made." Why is it appropriate to only consider the described subset? Does the model with the thus determined parameter values also cover the kinetics of the other pseudopods?

Technically it is practically impossible to incorporate cells that make pseudopods both in the Old and New pseudopod, because data analysis becomes extremely complicated. This is explained in the revised manuscript on page 12.

____

Reviewer #2: The author describes a short- and a long-term memory in amoeboid cells. The short-term memory stores the information where the last pseudopod was formed for circa 20 seconds and is mediated by branched F-actin and the SCAR/WAVE complex. The long-term (2 min) memory depends on a cGMP-binding protein that established the cell rear by inducing the formation of myosin II filaments. This inhibits the pseudopod formation at the rear and promotes the formation at the front.

Major concerns:

I find it difficult to follow up the neutrophil, mesenchymal and B.d. chytrid data, because the paper is not accessible for me at the moment. It comes in the last paragraph a little bit out of the blue. The author could explain it a bit more why he has chosen those. It seems that neutrophils and Dicty are fast, while Bd and mesenchymal stem cells are slow. It is PLOS so he can write as much as he wants.

The other paper is now available as PLoS ONE 15(12): e0243442. I asked the journal to send the current manuscript to the same editor and referees of the previous manuscript, but apparently that was not possible or did not happen. I have added all relevant basic information in the method section so the reader does not have to read the other paper.

Scar S55D phospho-mutant – Why does in particular this mutant does not split pseudopods (alternating left and right) like wild-type cells? I found their JCS paper, but that also does not tell me more. It would make reading easier with a bit of background. They refer to a Current Biology paper, but the description of the mutants does not agree, neither is there anything on dictybase. Or

is it S5D from Ura et al? Since it is the only mutant he looked at, that has problems with memory positioning it would be important to know. It appears to be in the SHD domain.

I thank the reviewer for this remark. The mutant we named Scar-S55D is the same as the mutant described as SCAR-SD by Ura et al in Current Biology. We made a mistake in handling the mutant and gave it a wrong name. I have carefully tried to reconstruct what has happened and come to the following. The mutant SCAR-SD has five serines mutated to aspartates. The mutant was received from the Insall lab most likely with the name SCAR-S5D (or SCAR-5SD). This was read as SCAR-55D and as such entered in our stain database. Since it is a phophomimetic mutation is was misunderstood as Scar-S55D. Amino acid number 5 and number 55 are not serines, so the name S55D is obviously wrong. In the revised manuscript Scar-S55D is replaced by SCAR-SD, and a remark is made in the method section on the wrong name in our previous JCS paper.

The interpretation of the results with a model of SCAR action (page 20/21) was not affected by wrong name.

It is essentially not new data, but I like the quantification he has done and how he combined the long and short memory story to explain directed movement. So good enough for this type of submission. To measure the angles is a good way to access forward movement using pseudopod splitting.

Data analysis is done carefully and the timely separation of the activation of ras, rap1 etc. is quite impressive. The data quality looks good and is convincing for me. Like the turning experiments, too. The finishing model is also a good summary.

Thank you.

Minor concerns:

Maybe call it SCAR/WAVE, and reference accordingly, then maybe also people outside the Dicty community look at the paper?

Very good suggestion. Scar has been changed to SCAR where specifically the *Dictyostelium* protein is meant, and to SCAR/WAVE at all other places.

The switching between left and right, North and East to describe pseudopod formation is a bit confusing. It is sometimes difficult to follow if developed or vegetative cells have been used for the analysis.

North has been replaced by ‘0 degrees’ and east by ‘90 degrees’.

Page 3:
bF-actin – branched F-actin
cGMP binding protein – name it here directly gbpC?
… extend pseudopods in the rear 70% of the cell, because ... - … extend pseudopods in the rear (70% of the cell), because... (make it easier to read)
Done.

Page 4:
Scar/Factin – SCAR/WAVE/F-actin
Done.

Table 1:
Are forAEH, racE and rap1G12V cells starved or vegetative? What is the purpose of the blue arrow in the last column connecting scar S55D vegetative and starved?

forAEH, racE and rap1G12V are starved cells, which is now indicated in revised manuscript. The blue arrow was to indicate that persistence of starved and vegetative SCAR-SD are significant different. Blue arrow is deleted in revised manuscript and the difference indicated by *** and explained in the legend.

Figure 7:
The capitalisation of Old and New looks a bit odd and unnecessary.

Without capitalization the text becomes difficult to read. A New axis is an entity that begins as a new axis, but remains the New axis in the experiment. Similarly, when a new axis appear, the current axis becomes the Old axis for the rest of the experiment.

Reviewer #3: This is a nice work demonstrating the ordered pattern of successive pseudopod extension demonstrating a positional memory (pseudopod P3 remembering the position of pseudopod P1 and anticorrelated with pseudopod P2…) and how it is related to the polarity axis memory and to the regulation of the acto-myosin machinery.
The experiments suggest that a lower pseudopod activity in the front is the input signal to generate a new (de novo) pseudopod at a random new location, and hence modifies the polarity axis. The mechanism proposed in the discussion (Fig. 9) with the role of the signaling pathways with Ras-GTP and Rap-GTP in the regulation of this polarity are convincing as well.
The paper is well written in particular the introduction.

One problem with this paper is the fact that the majority of pseudopod data are identical to those used in ref [15 = van Haastert, P.J.M. (2020). Unified control of amoeboid pseudopod extension

in multiple organisms by branched F-actin in the front and parallel F-actin/myosin in the cortex. Submitted].
This reference is not deposited on a reprint server so it is difficult for me to evaluate the novelty of the current work, and especially to evaluate raw data for the biophysical part I can better judge. For instance, I would like to watch movies but none is included here.

As mentioned to reviewer #2, the other paper is now available as PLoS ONE 15(12): e0243442. I asked the journal to send the current manuscript to the same editor and referees of the previous manuscript, but apparently that was not possible or did not happen. I have added extra basic information so the reader does not have to read the other paper.

The two papers make use of the same pseudopod data, but analyze this in very different ways. The previous paper aims at analyzing the dynamics of pseudopod extension; the data used deals with the timing and probabilities of the time of start and stop of pseudopod extension, leading to a unified model for the kinetics of pseudopod extension. The current manuscript aims at defining memory that is associated with persistence; the data used deal with the position in the cell where pseudopods are formed, leading to the recognition of short term and long term memory.

Table S2 of the revised manuscript indicates the source of the movies used in the previous and current manuscript.

Mean squared displacements (MSD) fits are used to fill the third column of Table 1 (persistence time) and these important data are discussed at several occasions. The origin of the MSD data (except a short example given in Fig. 1B) is not discussed. Is it from the same submitted ref [15]? The author should present all raw MSD curves (11 conditions=11 panels) with fits in supplementary information to evaluate their quality.
Usually reliable MSD analysis need a lot of statistics: either very long trajectories (up to 20h) as in Li and Cox (PLoS ONE 2008) or very numerous cell tracks (several hundred lasting about 1 h) as in Gole et al. (PLoS ONE 2011). I am surprised that sometimes there is only 8 cells analysed at 1 frame/sec making difficult to record long enough trajectories to analyze rigorously their persistence time.
In the same line of argument, how is calculated the error on the fitted persistence time in Table 1?

The reviewer is correct that long trajectories are needed for accurate fitting of MSD data to obtain accurate estimates for persistence and step size. The data are a sequence of 30 to 40 pseudopods per cell. All data have been thoroughly re-analyzed and described in new paragraphs on pages 25/26 of the method section and page 14/15 of the results. Briefly, long synthetic trajectories were generated that were analyzed by MSD as follows. First, long trajectories were generated from multiple cells of a specific strain by concatenating the pseudopod trajectories such that the first pseudopod of the next cell in the concatenate start at the same position and in the same direction as the last cell of the previous cell. These trajectories of >250 steps were analyzed by MSD, yielding estimates for the step size and persistence. The confidence of the estimates was determined by bootstrap analysis as indicated on page 26 of the revised manuscript. The raw MSD for all 11 strains and the obtained estimates are presented in a new supplementary figure S4 and table S1.

Page 19, I am not convinced by the conclusion that pseudopod P3 is slightly biased toward P2 when P2 direction is far from P1 direction. This conclusion holds from a better non linear fit with two fitting parameters than a linear one with one fitting parameter. But it is a general rule that the more fitting parameters, the better the fit !

The reviewer is correct: more parameter always lead to a better fit of the data. To test which model fits the data statistically significant better the Akaike Information Criterion (AIC_c) was used that gives a penalty to the number of parameters used in the model (see page 26). The model with the minimal AIC_c value is regarded as the optimal model. For the model with one parameter $AICc_1 = 4841$ and with two parameters $AICc_2 = 4832$. Added to the revised manuscript is the following: The quantity $\exp((AICc_1 - AICc_2)/2) = 90$ indicates that the model with two parameters is 90 times as probable as the model with one parameter.

Looking at the vertical scatter of raw data for a given ph1,2, the ratio between the black dot scatter width and the green bias width is at least 2 ! One possibility would be to estimate the error bar on the two parameters of the non linear fits to learn how reliable is this parameter. Perhaps there is a more systematic procedures to evaluate the significance of the proposed fits.

See above.

Minor comments.

A missing dot page 6 after [6,7,11]

Correction made

Explanation of the first order kinetics is unclear (data of Figs. 4b,e, introduced in page 13). Why x-scale in 4B goes only to 12s, not 25s as in 4a ?

The manuscript does not explicitly provide kinetic schemes with first order reactions. Therefore, in the revised manuscript the kinetics is explained descriptive: The kinetics of learning exhibits an exponential approach to equilibrium (Fig. 4ab) and exponential decay (Fig. 4de). In 4B the x-scale does not go beyond 12s, because equilibrium is nearly reached at 12 s.

Page 4: Don't we expect a lower %LR for vegetative than polarized cells ?? Please comment.

I understand the confusion, because vegetative cells have reduced splittings, and therefore %LR is expected to be reduced if related to all pseudopods. However, %LR only refers to sequential splitting pseudopods. Therefore, in the revised manuscript %LR is defined at the beginning of the paragraph (The fraction of sequential splitting pseudopods that are extended alternatingly to the left and right (%LR)….) and the sentence on vegetative has been rewritten: Although vegetative unpolarized cells do not extend many splitting pseudopods, the cases with sequential splittings have a high %LR.

The pseudopods intervals (in s, or min) for neutrophils, mesenchymal stem cells and B.D. chytrid should be indicated in Table 1.
I assume these cells have different intervals but the same persistence time in pseudopods number.
I feel this can be better discussed in the paper.

The pseudopod intervals of all cell lines is now mentioned in the new table S1. It shows that mesenchymal stem cells have extremely long persistence time, due to the extreme large pseudopod interval.

On the model page 18: I don't understand the sentence "Simulations reveal that phi depends predominantly on the kinetic parameters a and b". -> Which simulations ? -> What about calculating and plotting, phi as a function of Pn, once parameters a and b are calculated with the current hypothesis that phi is independent of Pn and T ? This is a kind of self consistent-argument.

It was meant to explain that phi is not strongly dependent on T and Pn, but mainly on a and b. This was observed in calculations of the value of phi with the observed values of T and Pn and different values of a and b. This was called ‘’simulations’’ but ‘’calculations’’ is much better. This has been changed and better explained in the revised manuscript on page 12.
