# Supplementary material for: Short- and long-term memory of moving amoeboid cells
Source: PLoS One. 2021 Feb 11;16(2):e0246345. doi: 10.1371/journal.pone.0246345 (PMC7877599; doi:10.1371/journal.pone.0246345)
Supplement: Attachment — Submitted filename: Rebuttal letter.docx [file pone.0246345.s009.docx]

# *Rebuttal letter*

Dear Thierry,

Thank you for handling this manuscript and the possibility to submit a re-revised version of the manuscript.

I have followed the suggestions of the reviewer. Major changes are the new figure S5 showing tracks with pseudopods for four cell lines, and detailed information on autocorrelation.

The re-revised manuscript (marked track changes) has the changed annotated yellow for large pieces of changed text or text with equations, and as track-changes for small pieces of text.

The author received no specific funding for this work.

I hope that the manuscript is now acceptable for publication in PLoS One. With kind regards,

Peter van Haastert

***Response to the reviewer***

Reviewer #3: The author has changed significantly the paper taking into account many of the reviewer’s comments including mine. It is much more clear now.

However, I feel some important points should still be considered and I cannot recommend this work in its present state.

1) It is very valuable to have access to all raw MSD (new Fig. S4). Generally the quality of the data set seems reasonably good to some exceptions: (i) the solid and dashed lines are very far at both short and long time steps for the starved gbpc-null data (part 2). (ii) The mesenchymal and the B.d. Chytrid data are not very well fitted by the Furth model at any time scale as well (Eq. 1): the data alternate above and below the Furth model and I am surprised by the low error on the fitted parameter. For instance, if we fit the data between n=0 and n=50 instead of 75, I am sure the asymptotic P value will be lower than the range indicated in Table S1. The author uses a bootstrap analysis (without detailing it) due to the non linear nature of the model. I will be curious to compare the resulting error on P with another method using a linear asymptotic fit (dashed line, so fitting between for instance n=30 and 50/60/70/80 for a comparison) and a 95% confidence interval method.

In this manuscript, persistence was analyzed to understand the consequence of memory for cell movement. Since movement and memory of movement were analyzed for pseudopod steps rather than distance of the cells centroid moved in time, it is essential to analyze persistence in steps as well. So equation (1) was written in a form with steps (see also reference 10), and the experimental data were analyzed accordingly to get an estimate of the persistence in number of steps. The aim of the manuscript is not to analyze and describe persistence in detail, and evaluate difference aspects that can influence persistence such a step size, waiting times, etc. It is clear from table 1 that the two forms of memory both contribute to persistence. For the present manuscript that conclusion is sufficient. More details are distracting.

Having said that, the analysis must be correct. The 80 data points were analyzed with the non-linear equation (1) to find the values for the parameters labda and P yielding the lowest RSS. The confidence interval was analyzed with the well accepted bootstrap method for non-linear relationships. Reference 52 describes in very large detail how to do that in Excel. As described in the manuscript, the principle of bootstrapping is to analyze repeatedly independent replicates of the original dataset; a replicate is obtained by randomly selecting 80 data points from the original 80 data points (then in a specific replicate, obviously, some data points are not represented and others may be represented twice). As described, I made 30 replicates, analyzed all using the minimal RSS method yielding 30 estimates for labda and 30 estimates for P. Then the 95% confidence interval of these 30 estimates is reported in the table; the 95% confidence interval is defined as 1.96*SD of these 30 estimates. Small changes were made on page 26 to better explain bootstrap analysis.

A mistake was made in the figure for *gbpC*-null (in excel the data were fitted correctly, but a wrong link was made for the values for P and labda, leading to incorrect lines in the figure and incorrect data is Table S1). All have been corrected in the revised manuscript.

Not sufficient data were available or have been analyzed to discriminate between different aspects of persistent movement of mesenchymal cells or Bd cells to support or reject the Furth model. This was not the aim of this study, data are sufficiently accurate to estimate the persistence.

Obviously making the dataset smaller, e.g. ,0-50 will yield other estimates of the parameters, that may well be outside the confidence interval obtained for the larger dataset of from 0-80.

Fitting the linear part of the dataset is not very uncommon to obtain persistence values, but warnings in the literature mention that this easily leads to underestimation of persistence. For your information, I report below the linear regression with 95% confidence interval of the starved wild-type data between n=30 and n=50/60/70/80. But also between n=30/40/50/60 and n=80. The general observations are that linear regression underestimates the value of P, and overestimates that accuracy.

| interval | P (steps) | |
| --- | --- | --- |
|  | value | 95% CI |
| ***non-linear regression*** |  |  |
| n = 0 to 80 | 14.902 | 0.960 |
| ***linear regression*** |  |  |
| n = 30 to 50 | 11.869 | 0.269 |
| n = 30 to 60 | 12.337 | 0.172 |
| n = 30 to 70 | 12.457 | 0.108 |
| n = 30 to 80 | 12.679 | 0.110 |
| n = 40 to 80 | 13.125 | 0.119 |
| n = 50 to 80 | 13.218 | 0.228 |
| n = 60 to 80 | 14.228 | 0.484 |

2) The Eq (1) is a Furth formula written is an unconventional way with lambda the step length. Usually, one uses the diffusion constant D or the speed V or the persistence length d= P lambda = V tp where tp is the persistence time (in s or in min, actually refered as “P, time” in min in Table S1). I recommend a more conventional form in addition to the one given by the author with his step unit (Eq 1).

The notation < ,+ ^2 > has to be changed as the index “i” is an averaged variable, and the only remaining variable is n which is the time t in steps, n=t/int (where "int" is the interval reported in Table S1). Do not use capital D which is usually reserved for the diffusion constant (which is important to be reported as well, see my next comment). I suggest for instance r^2(n) or MSD(n).

(^ means exponent)

As mentioned above, the experiments do not measure displacement as a function of time, but as a function of the number of pseudopods made. Time *t* is present in the data only indirectly through the average pseudopod interval. Therefore MSD of the primary data is a function of *n*. The equation has been changed to the mean-square displacement as a function of n, MSD(n).

2) The diffusion constant D=(V^2 tp)/2=(P lambda^2)/(2 int) for each cell line should be reported in Table S1 in µm^2/min.

By looking at the data, I am calculating D=275 µm^2/min, 81 µm^2/min and 63 µm^2/min for starved Dd, vegetatitive Dd and neutrophils respectively. I am a bit surprised by the low value for the neutrophils which in my mind was even faster than Dd. The author should compare their value with literature data.

The reviewer is correct that the data provide information on the diffusion constant. I prefer not to go into much detail here for two reasons: Firstly, persistence and the method of cell movement is not the primary aim of the manuscript. Secondly, neutrophils move faster than Dicty in a chemotactic gradient, but slower in buffer, which is also not the subject of this manuscript.

3) For the other organisms investigated, the only reference to the source of the movies in the new Table S2 is a bit short for this reviewer. I cannot evaluate without seeing the raw data (and these raw data are not present as well in the companion PLoS ONE paper recently published by the author) if the same concept of polarity and memory, with rear, front specificities is explaining convincingly their crawling motion.

It will be important at least in SI to show some trajectories with the alternating zig-zag front protrusions such as the one of Fig. 1a for these neutrophils, mesenchymal or Bd Chytrid cells. Finally, please show how the algorithm Quimp3 is detecting protrusions for these organisms.

The movies can be obtained using the reference given in Table S2. I have not made these movies myself, so detailed information on how cells were grown , etc. can either be found in the referenced manuscript or from the author of that movie, for which links were given in table S2. Trajectories are given in the revised manuscript Figure S5. These cell lines, as all cell lines, were initially analyzed by both quimp3 and the semi-manual method as described in the paper. High congruency of the two methods was obtained with the standard settings of quimp3 for Neutrophils, slightly modified setting for curvature for Bd, while quimp3 was not effective with mesenchymal cells due to the very spiky appearance of the pseudopods. This is mentioned in the revised manuscript on page 25.

4) Experimental conditions are missing with these other organisms: cell types (primary cells or cell lines like HL60...), recording conditions, composition of the culture medium...etc ? These information are not

present as well in the other PLoS ONE paper.

See above.

4) I am not satisfied with the response to my comment:

“Page 19, I am not convinced by the conclusion that pseudopod P3 is slightly biased toward P2 when P2 direction is far from P1 direction. This conclusion holds from a better non linear fit with two fitting parameters than a linear one with one fitting parameter. But it is a general rule that the more fitting parameters, the better the fit !

Looking at the vertical scatter of raw data for a given ph1,2, the ratio between the black dot scatter width and the green bias width is at least 2 ! One possibility would be to estimate the error bar on the two parameters of the non linear fits to learn how reliable is this parameter. Perhaps there is a more systematic procedures to evaluate the significance of the proposed fits.”

The author answered:

“Using the Akaike Information Criterion (AIC) the polynomial model describes the experimental data 90- times better than the linear model (see legend Fig. 8a).”

I am not able to evaluate that answer but I really feel suspicious about this magical mathematics. Does a model with a third parameter fit 900-times better the data ? I suggest the editor to ask some specialist in statistics to evaluate this statement.

The Akaike Information Criterion (AIC) is a standard method to evaluate models with different numbers of parameters. The parameters are fitted to the data using minimal RSS method. Obviously more parameters fit better. Therefore AIC in its most simple form has the format of AIC = Nln(RSS)+2p, where N is the number of observations and p is the number of parameters. The model with the lowest AIC value is the preferred model, so p is a penalty with the implication that RSS must improve substantially at higher numbers of parameters to obtain a lower value of AIC. (I have used the slightly different AICc that for other experiments in the manuscript is better at low N). For your information, I have fitted the data with three parameters (y=ax +bx^3+cx^5), yielding nearly the same RSS and higher AIC; see table below for p=1, 2 and 3. Clearly p=3 is over-fitting the data.

| number of parameters | RSS | AIC |
| --- | --- | --- |
| 1 | 4.027*10^5^ | 4841.7 |
| 2 | 3.910*10^5^ | 4832.7 |
| 3 | 3.907*10^5^ | 4834.4 |

Minor comments.

The autocorrelation of Fig 3b is not defined: please define it and explain as well how is estimated the pseudopod interval as these two quantities are compared.

The Autocorrelation function is now defined on page 25 of the revised manuscript. Pseudopod intervals were derived from the reference indicated in the table S1.

Fig 4: what is the difference between dark and gray bullets ?

The dark filled bullets were incorporated in the linear regression of panel 4e; this is indicated in the legend of the figure in the revised manuscript.

Fig S4, part 1, there is two “Dictyostelium” graphs (I guess starved WT ) but next page in Fig S4/part 2 there are again "starved WT" data presented.

Fig S4 part 1 has for comparison Dictyostelium staved WT and three other cell lines, and parts2/3 have for comparison the same Dictyostelium staved WT and mutants. This is now indicated in the legend to the figure.
